# Evaluation of Bacterial Contamination in Donated Blood for Transfusion Purposes at Kisii Teaching and Referral Hospital

**DOI:** 10.1155/ah/6934791

**Published:** 2025-06-28

**Authors:** Collince O. Ogolla, Rodgers N. Demba

**Affiliations:** ^1^Department of Applied Health Science, School of Health Science, Kisii University, Kisii, Kenya; ^2^Department of Medical Laboratory Science, School of Medicine, Maseno University, Maseno, Kenya

## Abstract

**Background:** Bacterial contamination of donated blood has been a major public health problem. It poses grave risks to the recipient.

**Objective:** The objective of this study was to determine bacterial contamination in donated blood for transfusion purposes at Kisii Teaching and Referral Hospital.

**Methodology:** This was a cross-sectional study. Sample collection was performed in BD BACTEC culture bottles and analyzed by BD BACTEC Machine FX40 for the presence of bacteria and thereafter subcultured for the positive vials. Biochemical tests were performed followed by confirmation tests with API-20 to identify bacterial presence. Samples negative for bacteria were not subjected to further analysis, and the results were directly recorded. Quality control procedures were performed using known ATCC microorganisms (*Staphylococcus aureus* [*S. aureus*] ATCC 25923). The data were entered into Excel and analyzed by SPSS Version 25.

**Results:** The general prevalence of bacterial contamination was 21.3% (23/108). The blood group A positive had the highest contamination rate (10.2%), while the prevalence by age was also higher in the 21–30 years age group (24%). The most commonly isolated organisms were *Staphylococcus epidermidis* (*S. epidermidis*) (56.5%), *S. aureus* (39.1%), *Bacillus* spp. (30.4%), and *Escherichia coli* (*E. coli*) (17.4%). Logistic regression analysis indicated that blood group A positive individuals (OR = 2.5, *p*=0.02) and the 21–30 years age group (OR = 1.8, *p*=0.03) were significantly related to contamination at odds. The association of blood group A positive and age 21–30 further augmented this risk (OR = 3.5, *p*=0.01).

**Conclusion:** *S. epidermidis*, *S. aureus*, *Bacillus* spp. and *E. coli* were among the isolated and identified bacteria found in donated blood samples among donors at KTRH.

**Summary**

• What this study adds?
∘ This study provides data which may be used to develop proper guidelines for the prevention and management of bacterial contamination of donated blood.

## 1. Introduction

Bacterial contamination of donated blood is a major global public health issue that can cause serious complications in transfusion recipients [1]. Such complications may include sepsis and, in some cases, death, especially with the transfusion of blood components such as platelets. Platelets, being stored at room temperature, are highly prone to bacterial contamination, while red blood cells (RBCs), fresh frozen plasma (FFP), and cryoprecipitate are less susceptible due to their specific storage requirements [1]. However, bacterial contamination in these components, though rare, remains an important concern. Common pathogens implicated in transfusion-transmitted bacterial infections include Gram-positive bacteria such as *Staphylococcus aureus* (*S. aureus*) and *Staphylococcus epidermidis* (*S. epidermidis*), as well as Gram-negative bacteria such as *Escherichia coli* (*E. coli*) and *Pseudomonas aeruginosa* [2].

Blood donation and blood product contamination can occur due to a study that indicates several parameters. The parameters include suboptimal skin preparation during venipuncture, poor collection of blood bag, and donor bacteremia [3]. Moreover, growth of the bacteria in the blood may remain elusive visual signs with the contaminated units that may not express overt signs including color change or smell [4]. While several measures to ensure safety are working against it, the blood remains at stake for contamination arising with an occasional estimate of 0.1%–0.2% in developed countries, for instance, in the Unites States and France [5]. On the other side of the scale are developing countries expected to suffer higher rates, with Kenya showing an 8.8% contamination rate [6].

The study was conducted in Kisii Teaching and Referral Hospital (KTRH), Kisii County, Kenya, and it focuses on bacterial contamination in blood donations meant for transfusion. This study is expected to fill the gap by contributing to the available information on contamination rates in the area. The study specifically investigates the types of bacterial species in donated blood, their frequency of occurrence, and possible correlations with demographic characteristics such as age, sex, and blood group. Also, the results can guide the creation of interventions aimed at reducing the possible risk of contamination, thereby improving transfusion safety within Kenya.

Identification of bacteria was investigated using the BD BACTEC culture system for bacterial detection followed by subculture for biochemical tests and specification of isolated pathogens using API-20 kits. The findings are expected to provide some useful information on the bacterial contamination of blood donations at KTRH, which will in turn allow the formulation of local guidelines to minimize contamination and improve blood safety protocols.

## 2. Methods

### 2.1. Study Design

A cross-sectional design was used to assess bacterial contamination in donated blood for transfusion at KTRH, Kenya. The cross-sectional design provided a brief assessment of contamination rates at a specific point in time, providing good insight into the prevalence and types of bacterial pathogens present in blood donations.

### 2.2. Study Site

The laboratory department of KTRH was the study setting, which is among the larger referral and teaching hospitals in Kisii County. The hospital is located in Kisii Town, Western Kenya, at an altitude of 1660 m above sea level.

### 2.3. Ethical Considerations

Ethical clearance was granted by the Baraton Ethics Review Committee (Ref: UEAB/ISERC/02/05/2022) for this study, while a research permit was conferred by the National Commission for Science, Technology and Innovation (NACOSTI), permit number NACOSTI/P/22/17542. All participants gave written informed consent after the study objectives, procedures, and potential risks were clearly explained to them. Participation was voluntary, and all participants were assured confidentiality and security concerning any information pertaining to them.

### 2.4. Participants and Inclusion/Exclusion Criteria

Blood donors who had volunteered in the KTRH blood transfusion center were the subjects of the study. Inclusion criteria were adult participants (≥ 18 years) who were willing to give informed consent and meet eligibility criteria for blood donation. Exclusion criteria concerned refusal for consent or a recent history of infection, chronic disease, and antibiotic use within the preceding 2 weeks, as these may introduce bias in the contamination analysis.

### 2.5. Sample Size Calculation

The sample size was calculated using Krejcie and Morgan's formula for finite populations, assuming the total population of 150 voluntary donors visiting the hospital in January 2022. A calculated sample size of 108 was thus obtained with a corresponding 95% confidence level and 5% margin of error for statistical reliability.

### 2.6. Sample Collection and Handling

Blood samples from donors were collected aseptically into BD BACTEC culture bottles to minimize the chances of contamination during venipuncture. There was a standardized method of disinfecting the skin to avoid the introduction of bacteria during blood collection. Blood samples were collected by trained phlebotomists using 16-17 gauge needles, ensuring that no form of contamination entered the blood sample, the blood bag, and surrounding materials.

### 2.7. Bacterial Detection and Identification

The blood sample collected was immediately processed in the BD BACTEC FX40 Automated Blood Culture System. This system works on principles of fluorescent technology for bacterial detection in blood culture bottles. Leveraging fluorescence technology, this system detects the growth of bacteria in blood culture bottles, with positive samples confirming bacterial growth through the subculture on other agar media. Such media included several subcultures such as nutrient agar: this nutrient biotechnology allows general purpose for initiation of bacterial growth; MacConkey agar: it is selective for Gram-negative bacteria with emphasis on Enterobacteriaceae; eosin methylene blue (EMB) agar: it differentiates to Gram-negative bacteria using the differential method, thus, it can help in identifying *E. coli; and* blood agar: it helps in assessing hemolytic activities and identifying *S. aureus* and other hemolytic bacteria. After subculturing, bacterial colonies were Gram-stained to reveal the morphological characteristics. Further testing involved biochemical tests such as catalase, coagulase, and oxidase tests to identify bacterial species. The API-20 System, a standardized method for species identification based on biochemical reactions, confirmed the bacterial species.

### 2.8. Quality Control

Quality control measures were used throughout this study to guarantee the accuracy of the study. Control of the bacterial identification process included application of known ATCC microorganisms such as *S. aureus* ATCC 25923. The entire sample handling processes from collection to analysis were also carried out with stringent sterility conditions to avoid contamination.

### 2.9. Statistical Analysis

Data collected were entered into Microsoft Excel spreadsheets and subsequently analyzed using SPSS Version 25. Data were summarized using descriptive statistics. The chi-square test was used to determine the association between bacterial contamination with respect to age, sex, and blood group. A *p* value of less than 0.05 is considered statistically significant. Data were visualized through charts and tables for easy interpretation and presentation of results. Multivariate analyses were considered to explore possible risk factors and their correlation with bacterial contamination.

### 2.10. Limitations

The methodology is well standardized for bacterial detection; however, there were some important limitations. First, since the study was cross-sectional, it only showed a momentary picture of bacterial contamination without showing any temporal trends or leading to conclusions. Second, the study relied on voluntary donors, hence possible selection bias. Nevertheless, it throws some valuable light on the prevalent types of bacterial contamination found in blood donations at KTRH hospitals.

## 3. Results

### 3.1. Bacterial Presence in Donated Blood

**Figure 1:**
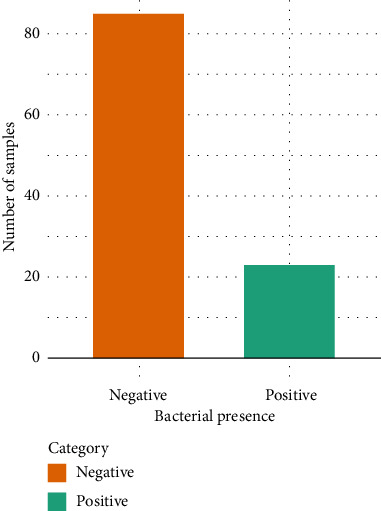
Bacterial presence in donated blood.

**Figure 2:**
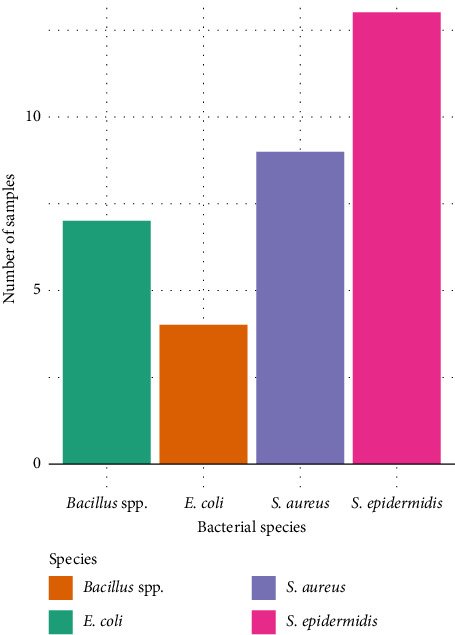
Bacterial species in contaminated blood.

The results from the analysis showed that 23 out of the 108 samples exhibited growth in the BACTEC bottles of the initial culture. This represented 21.30% of the total sample size. 85 out of the 108 samples did not exhibit any growth in the BACTEC bottle of the initial culture. This represented 78.70% of the blood donors who participated in the study, as shown in Figures 1 and 2. The BACTEC positive samples were further subjected to subculture to identify the bacterial presence.

**Figure 3:**
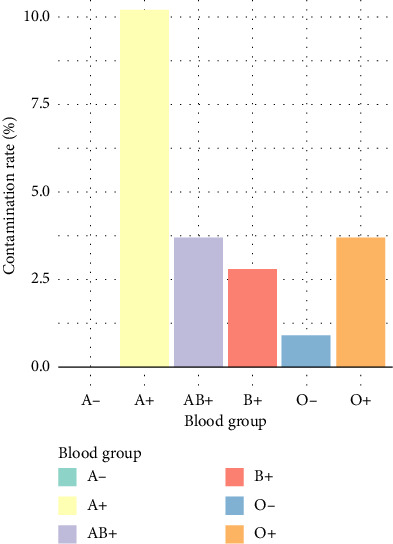
Bacterial contamination by blood group.

On a subculture of BACTEC-positive bacterial growth, there was growth of white circular colonies with smooth margins on the nutrient agar. Growth on mannitol salt agar was observed with round yellow and colorless colonies with smooth margins and an elevated surface. Pink colonies grew on MacConkey agar. EMB media exhibited growth with blue–black small round colonies and pink colonies. Colonies on blood agar exhibited hemolysis. A total of 23 of the 108 samples which turned BACTEC-positive for bacterial contamination were further analyzed and distributed according to blood group. Blood group A positive showed the highest prevalence of bacterial contamination with 11 samples which represented 10.2%. Blood group O positive and AB positive had the same distribution of 3.7%, followed by B positive which had 2.8%. Blood group O negative had the lowest positivity rate with only 1 having bacterial contamination which represented 0.9%. There was no bacterial contamination in blood group A negative, representing 0.0%, as illustrated in Figure 3.

**Figure 4:**
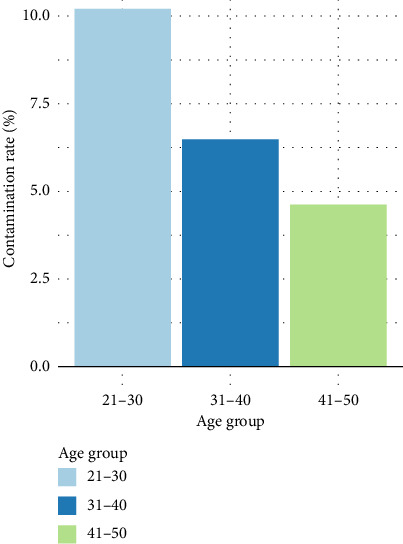
Distribution of bacterial contamination by age group.

Based on the results in Figure 4, the majority of the participants aged between 21 and 30 years showed a higher level of blood contamination with 10.19%, followed by 6.48% for those aged between 31 and 40 years and lastly 4.63% representing those aged 41–50 years (Figure 4).

On subculture, bacteria grown on media were identified by Gram stain, various biochemical tests, and API-20 as *E. coli*, *S. epidermidis*, *Bacillus* spp., and *S. aureus*. 12 out of the 23 samples representing 52.2% exhibited mixed contaminations. Four samples were contaminated with *E. coli* representing 17.4% of the contaminated samples. Nine samples, representing 39.1%, were contaminated with *S. aureus*. Seven samples, representing 30.4%, were contaminated with *Bacillus* spp. and 13 samples, representing 56.5%, were contaminated with *S. epidermidis*. The results of the study confirmed that the majority of the etiological agents for bacterial contamination of blood were Gram-positive bacteria; hence, the prevalence rate for specific bacteria was high in *S. epidermidis* with a count of 13 out of the 23 BACTEC-positive samples, representing 39.39%. Furthermore, *S. aureus* bacterium had a distribution of 9, (27.27%), for *Bacillus* spp. and *E. coli* had a distribution of 21.21% and 12.12%, respectively, as shown in Table 1.

**Figure 5:**
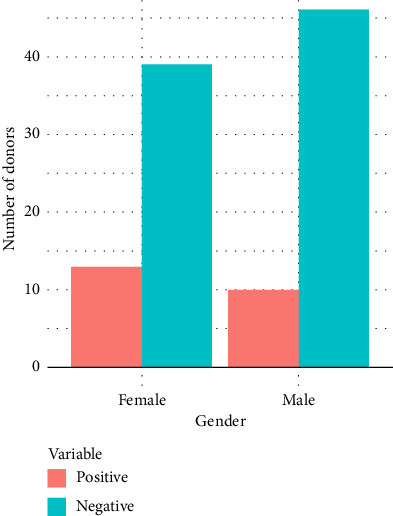
Gender influence on bacterial contamination.

From Table 1, a total of 23 positive cases of bacterial presence were detected, in which 10 (9.3%) were male and 13 (12.0%) were female, and a total of 85 negative cases were detected, that is, 46 (42.6%) for the male and 39 (36.1%) for the female. The chi-square test's statistical analysis result of 1 degree of freedom with an asymptotic significance value of 0.365 is greater than 0.05. This indicates that, there was no association (there was no influence) of gender on the bacterial contamination in donated blood for transfusion purposes as illustrated in Figure 5.

### 3.2. Multivariate Analysis of Factors Associated With Bacterial Contamination

A multivariate analysis was conducted to assess the independent contributions of blood group, age group, and others on the probability of bacterial contamination in blood donation. The logistic regression analysis had bacterial contamination (yes/no) as the dependent variable and blood group, age group, and other demographic variables as independent predictors.

### 3.3. Logistic Regression Model

**Figure 6:**
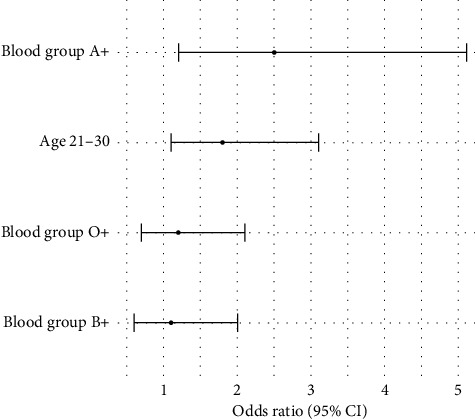
Predictors of bacterial contamination.

The logistic regression analysis showed that blood group (A positive) and age group (21–30 years) were good predictors for bacterial contamination in donated blood. The odds of contamination were greater for A-positive blood (OR = 2.5, *p*=0.02) and for donors aged 21–30 years (OR = 1.8, *p*=0.03), as shown in Table 2 and Figure 6.

### 3.4. Multivariate Analysis of Bacterial Species

**Figure 7:**
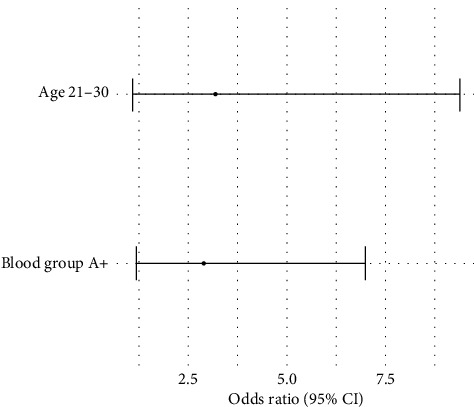
Predictors of *S. aureus* contamination.

The second multivariate logistic regression was conducted to identify the factors that influence the presence of specific bacterial species such as *S. aureus* and *S. epidermidis* in the blood sample from a contaminated source. Results indicated that *S. aureus* was isolated more from either the blood samples drawn from donors aged 21–30 years (OR = 3.2, *p*=0.04), or those found with blood group A positive (OR = 2.9, *p*=0.02). This implies that these factors are significantly correlated with the presence of this bacterial species. The detailed analysis is shown in Table 3 and Figure 7.

### 3.5. Interaction Effects of Blood Group and Age Group

**Figure 8:**
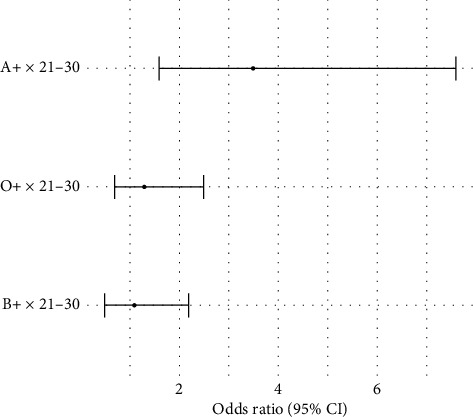
Interaction effects of blood group and age.

The interaction term of the blood group and age group was put into the logistic regression model to study the possible interaction effects on bacterial contamination. The interaction between blood group A positive and age group 21–30 years was significantly associated with bacterial contamination (OR = 3.5, *p*=0.01). This finding indicates that donors of this particular group (blood group A positive and age 21–30 years) are at a much higher risk of bacterial contamination than any other combination of blood groups and age. The analysis of interaction is presented in Table 4 and Figure 8.

### 3.6. Prevalence of Bacterial Contamination by Blood Group and Age Group

**Figure 9:**
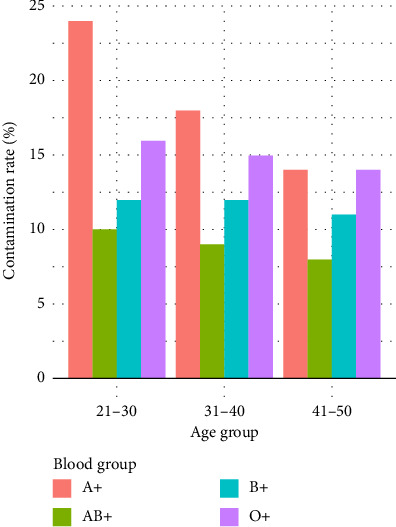
Contamination by blood group and age group.

An analysis further stratified by age and blood group indicated that blood group A positive donors aged 21–30 had increased bacterial contamination (24%) compared to all their other blood group–age combinations as shown in Table 5 and Figure 9.

## 4. Discussion

The present study assessed the levels of bacterial contamination in donated blood, focusing on the prevalence of several bacterial species and their possible association with factors such as blood group, age, and gender. The results concluded that the blood group A positive showed a higher prevalence of bacterial contamination than the other blood groups. These results corroborate the previous studies performed by the authors in [7], wherein it was noted that the A-positive group tended to be more prone to harvesting bacterial contamination. However, the study did not determine the mechanisms accounting for the observation. One possible explanation could be the predominant number of A-positive donors in this sample, rich in donations as some authors posited, where certain blood groups were found to have been overrepresented in blood donations [8]. Another explanation might relate to differences in immune responses among blood groups, but this requires further studies to clarify the actual variables impacting this trend.

Interestingly, the results from this study do not entirely corroborate the findings of other studies such as [9], which reported that individuals with blood group O positive had lower incidence rates of bacterial contamination than other blood groups. The differences may be due to variations in methodologies, geographical differences, or uncontrolled factors and either sample size or health conditions affecting the blood donors themselves. Hence, there is a need for further investigations into the correlation between bacterial contamination and blood group under a qualified set of confounding variables.

The 21–30-year age group had the highest prevalence of bacterial contamination (10.19%) compared to those in the 41–50-year age group, as observed in this study. Confirmatory results were by [5], who observed that blood donors less than 36 years might have shown more participation in blood drives due to relatively more enthusiasm and availability of these younger persons [10]. Higher bacterial contamination among younger donors may be argued to exist due to diverse skin flora, higher likelihood of disregarding sterilization procedures, or due to less careful handling during donations, but these will need further investigation to prove otherwise.

In terms of bacterial contamination, this study identified *S. aureus* and S. epidermidis as the predominant isolates, which is consistent with the findings of the authors in [11]. Both *S. aureus* and *S. epidermidis* are common skin flora, and the presence of these in the blood is an indicative sign of contamination at the time of collection and very likely because of failure in sterilization of the venipuncture site. The finding is also supported by previous studies where these species were the most frequently identified bacteria in contaminated blood samples prior to transfusion. The relatively easy access of CoNS into the blood culture samples, such as *S. epidermidis*, may have been caused due to poor cleaning methods and insufficient time given for disinfectant drying. Of the samples, isolates showed CoNS with predominance in 56.5% of samples, which corresponds with studies conducted in some African countries such as Nigeria [12] and Ghana [13]. This implies CoNS may pose a great risk for blood contamination since the preparation of the skin before venipuncture is a major challenge.

Poor disinfection of the site of blood collection is often cited as a major reason for contamination, since skin-related pathogens such as *S. aureus* and *S. epidermidis* predominate. Skin disinfection is important before venipuncture, and disinfection should start from the center and proceed outward with a radius of at least 2 cm. It is also desirable for the disinfectant to dry completely before blood collection, which usually takes not less than 30 s. Any mishaps during this procedure may jeopardize the actual bacterial contamination. Studies have also shown that alcohol and iodine pads work best if applied correctly and are given adequate time to work, as improper application can result in patches of skin being left inadequately disinfected.

This study had some limitations that need to be acknowledged. The first limitation is that the study considered bacterial contamination but not different forms of contamination, such as viral or fungal. A broad assessment would give a clearer understanding of the donated blood's overall safety. The second limitation is that, although the study demonstrated significant correlations between blood group and age with respect to bacterial contamination, the causal relationships remain unexplained due to the cross-sectional nature of the study. This is further complicated by the fact that selection bias, especially with respect to overrepresentation of blood groups (such as A positive) and younger age groups within the sample, is likely to inflate contamination prevalence estimates. Hence, in general, this might lead to overestimating the contamination risk among the wider donor population as a whole, especially if the sample does not fully represent the wider donor base. Such contamination patterns have to be combated by improving disinfection protocols which include application techniques, drying times for antiseptics (at least 30 s), and supporting staff training in aseptic methods. Future studies should explore the biological mechanisms involved in the relationship between blood type and bacterial contamination using prospective or longitudinal designs controlling for confounding variables. Further prospective studies with refined control measures will be necessary for further exploration of these associations.

## 5. Conclusion

This study provides undeniable evidence that bacterial contamination occurs with the highest percentage in blood donation and displays the two most common bacteria isolated in blood donations, i.e., *S.* epidermidis and *S. aureus*. Findings of blood group A positive and the age group of 21–30 years also revealed high-risk factors for bacterial contamination. However, the rates of contamination might be overestimated owing to their cross-sectional design and the kinds of selection bias that could include underrepresentation of some blood groups and overrepresentation of certain age groups, which in turn affects the overall generalizability of such results to the wider donor population. The recommendations designed to mitigate bacterial contamination and improve blood transfusion safety are as follows: (1) improve disinfection practices regarding venipuncture using appropriate techniques and allowing antiseptics to dry for at least 30 s and making regular staff retraining on strict disinfection practices; (2) increase staff training on aseptic techniques to reduce contamination during blood collection; and (3) prospective studies that investigate mechanisms linking blood group and bacterial contamination, which may give clues to biological or immunological factors affecting contamination rates. In summary, the study comes with a lot of information regarding risk factors of bacterial contamination; however, further research is needed to verify the causal relationships underlying the risks and to formulate proper donor safety protocols.

## Figures and Tables

**Table 1:** Chi-square test for gender influence on bacterial contamination in donated blood.

			Bacterial presence		Total	Chi-square test		
			Positive	Negative		Degree of freedom	Chi-square value	*p* value
Gender	Male	Count	10	46	56	1	0.821	0.365
		% of total	9.3%	42.6%	51.9%			
	Female	Count	13	39	52			
		% of total	12.0%	36.1%	48.1%			
		Count	23	85	108			
		% of total	21.3%	78.7%	100.0%			

**Table 2:** Logistic regression analysis of factors associated with bacterial contamination.

Factor	Odds ratio (OR)	95% Confidence interval	*p* value
Blood group A positive	2.5	1.2–5.1	0.02
Age group 21–30 years	1.8	1.1–3.1	0.03
Blood group O positive	1.2	0.7–2.1	0.29
Blood group B positive	1.1	0.6–2.0	0.59

**Table 3:** Logistic regression analysis of factors associated with the presence of specific bacterial species.

Bacterial species	Factor	Odds ratio (OR)	95% Confidence interval	*p* value
*Staphylococcus aureus*	Age group 21–30 years	3.2	1.1–9.4	0.04
*Staphylococcus aureus*	Blood group A positive	2.9	1.2–7.0	0.02
*Staphylococcus epidermidis*	Age group 41–50 years	1.4	0.7–2.8	0.31
*Staphylococcus epidermidis*	Blood group O positive	1.2	0.6–2.3	0.52

**Table 4:** Interaction effects of blood group and age group on bacterial contamination risk.

Interaction factor	Odds ratio (OR)	95% Confidence interval	*p* value
Blood group A positive × age group 21–30	3.5	1.6–7.6	0.01
Blood group O positive × age group 21–30	1.3	0.7–2.5	0.35
Blood group B positive × age group 21–30	1.1	0.5–2.2	0.72

**Table 5:** Prevalence of bacterial contamination by blood group and age group.

Blood group/age group	21–30 years (%)	31–40 years (%)	41–50 years (%)	Total contamination (%)
A positive	24	18	14	22.1
O positive	16	15	14	16.3
B positive	12	12	11	12.4
AB positive	10	9	8	9.8

## Data Availability

The data used to support the findings of this study are included within the article.
